# *Ligilactobacillus salivarius* PS11610 Enhances the Fertilization Success of IVF: A Preliminary Retrospective Analysis

**DOI:** 10.3390/nu17030410

**Published:** 2025-01-23

**Authors:** Miguel Raimundo, Patrícia Rodrigues, Sergio Esteban, Irene Espinosa-Martos, Esther Jiménez

**Affiliations:** 1 NOVA National School of Public Health, NOVA University Lisbon, 1600-560 Lisbon, Portugal; 2 School of Psychology and Life Sciences, Lusófona University Lisbon, 1749-024 Lisbon, Portugal; patricia.rodrigues@ulusofona.pt; 3 Probisearch SLU, 28760 Tres Cantos, Spain; sergio.esteban@probisearch.com (S.E.); irene.espinosa@probisearch.com (I.E.-M.); esther.jimenez@probisearch.com (E.J.)

**Keywords:** *in vitro* fertilization, probiotics, *Ligilactobacillus salivarius*, infertility

## Abstract

Background/Objectives: Infertility affects couples at reproductive age, with *in vitro* fertilization (IVF) being the most effective treatment. Success rates of IVF are influenced by several factors, including a healthy female reproductive system microbiome, which can improve implantation rates and pregnancy outcomes. This study evaluated the impact of *Ligilactobacillus salivarius* PS11610 on IVF outcomes. This strain showed antimicrobial activity against pathogens related to dysbiosis, commonly observed in women undergoing assisted reproductive treatment. Results: The administration of *L. salivarius* PS11610 at a dose of 1 × 10^9^ CFU every 12 h for at least one month before IVF procedures, particularly in the frozen embryo transfer (FET) group, appears to enhance the success rate of IVF. IVF procedures without embryo transfer showed no significant differences between the groups. However, there were statistically significant differences in the quality of embryos, specifically in category 2, which were higher in the group without *L. salivarius* PS11610 supplementation (*p* = 0.042). Similar results were seen in the IVF with embryo transfer group, where the quality of embryos in categories 2 and 3 was higher in the group without *L. salivarius* PS11610 (*p* = 0.019 and *p* = 0.05, respectively). IVF with FET showed notable improvements, where intake of *L. salivarius* PS11610 was associated with a significant increase in live birth infants (26.4% with *L. salivarius* PS11610 vs. 17.9% without, *p* = 0.034) and higher biochemical pregnancy rates (42.6% vs. 34%, *p* = 0.071). Conclusions: Despite some differences in embryo quality, the overall positive impact on pregnancy and birth outcomes highlights *L. salivarius* PS11610 as a promising supplement in assisted reproductive treatments.

## 1. Introduction

Infertility is defined by the World Health Organization (WHO) as the failure to achieve a clinical pregnancy after 12 months of unprotected sexual intercourse and affects 15% of couples worldwide [1].

The associated causes are divided, almost equally between male, female, or combined failure, leaving approximately 15–30% of infertility to unknown causes, also referred to as idiopathic [2].

Many infertile couples seek treatment options, with *in vitro* fertilization (IVF) recognized as the most effective method [3,4]. However, success rates of IVF cycles only lead to pregnancy with a live birth in about 30–35% of women [5,6]. Several factors can influence the success rate, including the embryo’s quality and the endometrium’s receptivity, including the microbiota of female reproductive system [7,8].

Human microbiota, the community of microorganisms living inside and outside our body, is a good indicator of our health status [9]. Until recently, this microbiota was evaluated in the context of disease, but this view is changing. In the last decade, numerous publications have broken the old paradigm that considered the urogenital tract as sterile, demonstrating that microorganisms present in the urogenital tract represent 9% of the whole human microbiome [10,11,12]. A healthy urogenital microbiome improves implantation rate and pregnancy outcomes, whereas 40% of dysbiosis prevalence is observed in women under assisted reproductive treatment (ART) [5,13,14,15]. A healthy intestinal microbiome was reinforced by studies suggesting improvement in disease outcomes such as colorectal cancer, inflammatory bowel disease, pancreatic ductal adenocarcinoma, and post-fecal microbiota transplantation [16,17].

The female urogenital microbiota is characterized by low bacterial diversity and *Lactobacillus* genus predominance (>90%) [14,16,17], protecting against pathogens by producing lactic acid, lowering the vaginal pH. They also prevent pathogens from colonizing by blocking adhesion sites on epithelial cells and producing substances like bacteriocins and hydrogen peroxide (H_2_O_2_) [18,19,20]. Recently, Moreno and collaborators showed better implantation rates in *In Vitro* Fertilization (IVF) in women whose endometrial microbiota was dominated by *Lactobacillus* [14].

Bacterial vaginosis (BV) is the most common urogenital dysbiosis, or bacterial imbalance in women, where *Lactobacillus* is usually replaced by a plethora of pro-inflammatory microorganisms such as *Gardnerella vaginalis*, *Atopobium vaginae*, *Prevotella* spp., and *Veillonella* spp. [18,21,22]. Although some women may be asymptomatic, BV induce vaginal itching, pain, or vaginal secretions, which may lead to new infections, and most importantly to infertility, and preterm birth or miscarriage, if occurring in pregnant women [23,24,25].

The urogenital microbiome also plays an essential role in spermatogenesis, the absence of Lactobacilli and the increase in *Prevotella spp.* was associated with alterations in semen quality, and hence male fertility [26,27,28,29].

It has been shown that oral or vaginal treatment with *Lactobacillus*, restores vaginal microbiota [9,30,31,32,33,34]. However, the direct effect of this supplementation on fertility and the endometrial microbiome needs more studies [35]. In males, treatment with Lactobacillus, was shown to improve sperm quality in almost every parameters [36,37,38].

Despite the studies using probiotic-based treatments, diseases associated with male and female urogenital bacterial imbalance are mainly treated with antibiotic therapy [32,39].

Fertibiome^®^ (Zinereo Pharma, Vigo, Spain) with *Ligilactobacillus salivarius* (formerly named *Lactobacillus salivarius*) PS11610 strain, is one of such probiotic treatments and it has shown its antimicrobial activity *in vitro* agains pathogens associated with bacterial imbalances of the female and male genital tract. In a pilot study, supplementation with *L. salivarius* PS11610 has shown to improve pregnancy and delivery rates by treating bacterial imbalance in 67% of couples [40,41,42].

The European Food Safety Authority (EFSA) has qualified *L. salivarius* as a microorganism with a Qualified Presumption of Safety (QPS) [43].

Considering the results obtained in the *in vitro* and *in vivo* trials, this study aimed to assess the impact of *L. salivarius* PS11610 on couples undergoing IVF treatments.

## 2. Materials and Methods

### 2.1. Study Population

Six hundred and ninety-four women aged 18 to 49 participated in their first IVF treatment, either with or without embryo transfer, at a fertility clinic in Lisbon, Portugal. This study was conducted over two one-year periods: from September 2021 to August 2022, during which no probiotic supplementation was administered, and from September 2022 to August 2023, when *L. salivarius* PS11610 (also identified as Fertibiome^®^) was included in the treatment protocol.

During the first period, women took only daily supplements prescribed by the gynecologist. During the second period, they took daily supplements prescribed by the gynecologist plus 1 capsule every 12 h of Fertibiome^®^ (10^9^ CFU of *L. salivarius* PS11610) for at least one month before the procedure.

The primary outcomes for women undergoing IVF in freeze-all cycles (no embryo transfer post oocyte collection) were the number of oocytes, number of frozen oocytes, number of fertilized oocytes, number and quality (1, 2, and 3, according to Istanbul Consensus classification) of embryos, number of frozen embryos, and number of blastocytes. For those women undergoing IVF with fresh embryo transfer, the primary outcomes were biochemical pregnancy (level of beta-hCG in blood > 5 and <50 IU/L), clinical pregnancy (value of beta-hCG in blood > 50 IU/L and ultrasound confirmation), miscarriage, and live births.

The data collected were oocyte origin (Own/Donor/Receiving Oocytes from the Partner (ROPA)), number of embryos transferred, biochemical pregnancy (No/Yes), clinical pregnancy (No/Yes), miscarriage (No/Yes), voluntary interruption of pregnancy (VIP) (No/Yes), ectopic pregnancy (No/Yes), heterotopic pregnancy (No/Yes), pregnancy type (Single/Multiple) and live birth (No/Yes).

In our study of IVF that does not involve embryo transfer, we gathered important data, which include: oocyte extraction procedure (Punction/Cryopreserved/Both), semen origin (Frozen Testicular Biopsy/Frozen/Donor/Fresh), fertilization procedure (IVF/Intracytoplasmic sperm injection (ICSI)), number of oocytes, number of cryopreserved oocytes, number of fertilized oocytes, number of embryos produced, number of blastocysts in each quality grade (1, 2, and 3), number of cryopreserved embryos, and number of blastocysts. In the IVF embryo transfer procedure, additional information was collected, including the following: the origin of oocytes (Fresh/Vitrified), the source of semen (Fresh/Frozen/Donor), the number of thawed embryos, the number of days from thawing to transfer, and the number of sacs.

These parameters were categorized based on their relevance to the procedure or the outcome, depending on the specific goals of the IVF process implemented (IVF without transfer, IVF with transfer, or IVF with frozen embryo transfer (FET).

### 2.2. Statistical Analysis

Data distribution was assessed using the Shapiro test, histograms, skewness, and kurtosis. Quantitative variables under normal distribution were summarized as mean ± standard deviation (SD), and comparisons between the two evaluated periods were assessed using a *t*-test. When data were not normally distributed, median and interquartile range (IQR) and the Kruskal–Wallis test were used. Qualitative variables were summarized using absolute and relative frequencies, and contingency tables were evaluated using the Chi-square test. Finally, multiple factor analysis (MFA) was used to summarize and visualize the relationship between characteristics and outcomes (quantitative and/or qualitative), considering the use of Fertibiome^®^ as the stratification factor. Results were considered statistically significant when a *p*-value equal to or below 0.05 was obtained. All the analyses were performed in RStudio 2022.12.0 + 353 “Elsbeth Geranium” Release (7d165dcfc1b6d300eb247738db2c7076234f6ef0, 3 December 2022) for Windows using R version 3.6.0 (26 April 2019)—“Planting of a Tree” Copyright (C) 2019 The R Foundation for Statistical Computing.

## 3. Results

### 3.1. IVF Without Embryo Transfer

From September 2021 to August 2023, a total of 148 cycles of IVF without embryo transfer were performed, of which 85 cases were without *L. salivarius* PS11610 and 63 with *L. salivarius* PS11610. The characteristics of the cycles are summarized in Table 1, and no differences between the two periods of time were found.

The procedure’s outcomes are summarized in Table 2. No differences were found for oocyte extraction, preservation, or fertilization features. Further, the number of embryos obtained, and their quality were similar in both periods except for embryos of quality 2, which were higher without *L. salivarius* PS11610 (*p* = 0.042 and *p* = 0.028, respectively).

### 3.2. IVF with Embryo Transfer

During the two evaluated periods, 76 cycles of IVF were performed, followed by embryo transfer: 44 without *L. salivarius* PS11610 and 32 with it. The characteristics of the cases are summarized in Table 3. Only embryos of quality 2 and 3 show some statistically significant differences between the two periods, with higher values in the period without using *L*. *salivarius* PS11610.

Regarding the outcomes, measured by biochemical (very early) and clinical (ultrasound confirmed) pregnancy, miscarriage, and live birth, as seen in Table 4, no statistically significant differences were found between with and without the intake of *L. salivarius* before IVF treatment (Table 4).

### 3.3. IVF with Frozen Embryo Transfer (FET)

**Figure 1:**
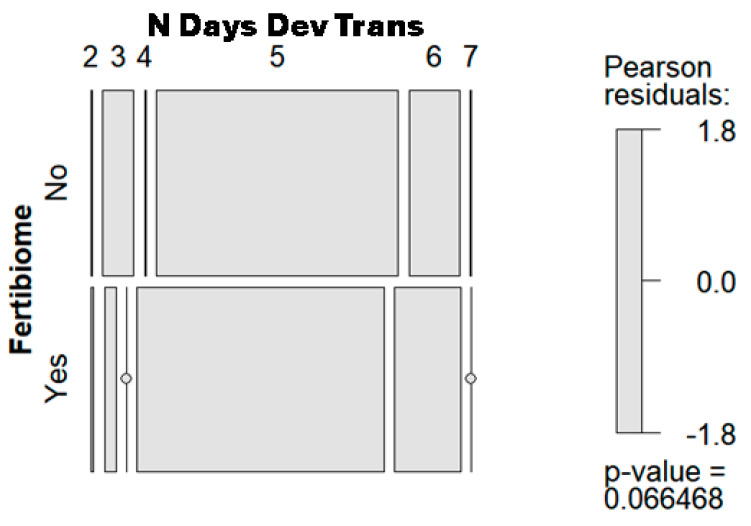
The mosaic chart of the distribution of the number of days from thawing to transfer, considering a multilevel factor, within both evaluated periods.

A total of 470 cycles of FET were performed, with 235 cycles in each evaluated period. Table 5 summarizes the characteristics of the cases, showing the similarity between the two periods being assessed. Only the number of days between thawing and transfer showed statistically significant differences between both periods when it was analyzed as a numeric variable, and also if the discrete values were considered as the level of a factor, using the Fisher test or the mosaic plot (Table 5 and Figure 1, respectively).

Regarding the primary outcome, the number of live births was higher during the period where *L. salivarius* PS11610 was intake compared to the one that it was not (62 (26.4%) vs. 42 (17.9%), *p* = 0.034). Biochemical pregnancies refer to early pregnancies that can be confirmed through a blood test detecting pregnancy hormones. The pregnancy may not yet be visible on an ultrasound at this stage. Biochemical pregnancies were also higher with *L. salivarius* PS11610 intake. However, this difference was statistically significant only at the 90% confidence level (80 (34.0%)/100 (42.6%), *p* = 0.071) (Table 6).

**Figure 2:**
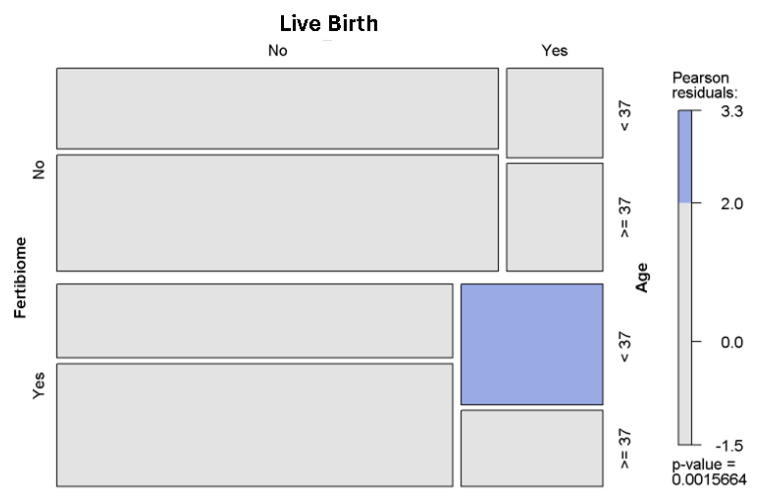
Mosaic chart of the distribution of live birth infants within the two evaluated periods according to age.

In Figure 2, the number of live births according to age is represented in a mosaic plot, which includes the Pearson residuals distribution and the signification of the log regression model. It shows a higher live birth rate in women under 37-years-old when *L. salivarius* PS11610 is used (*p* = 0.0016).

**Figure 3:**
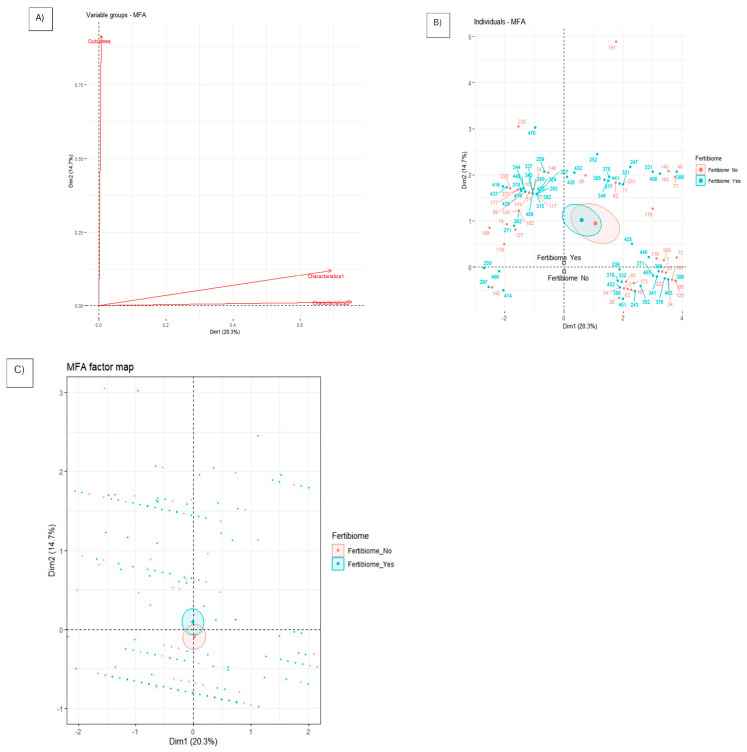
MFA chart of the groups of features represented with continuous arrows and labeled (**A**), MFA chart of the 95% confidence ellipses of the cases stratified by intake of *L. salivarius*—PS11610 (**B**), and MFA chart of the 95% confidence ellipses the stratified factor used (**C**) for the first two dimensions of the FET cases dataset.

This study’s dataset of IVF cycles with FET was organized into four feature groups. These included three qualitative groups: *L. salivarius* PS11610, qualitative case characteristics, and outcome characteristics. Additionally, one quantitative group focused on the quantitative characteristics of the cases. This organization was carried out to facilitate a Multiple Factor Analysis (MFA). *L. salivarius* PS11610 was used to stratify the cases but not to calculate the dimensions. The first two dimensions of the MFA explain the 35% of the variability with eigenvalues greater than 1 (Figure 3A–C). In the factor map, the groups of characteristics trigger the separation between cases in the first dimension, and the outcomes trigger the second dimension (Figure 3A). Regarding individual maps, there is no clear separation between the 95% confidence ellipses of the cases from both periods (Figure 3B). Nevertheless, a separation between the 95% confidence ellipses of the stratification factor “intake of *L. salivarius* PS11610” is shown, and this separation is almost due to the 2nd Dim, triggered by the outcomes evaluated (Figure 3C).

## 4. Discussion

The current study evaluated the impact of *L. salivarius* PS11610 on IVF procedures outcomes. The findings were analyzed across three groups: IVF without embryo transfer, IVF with embryo transfer, and IVF with FET.

The analysis of IVF procedures without embryo transfer showed no significant differences between the groups with and without *L. salivarius* PS11610 regarding the number of oocytes, fertilized oocytes, and embryos obtained. However, there were statistically significant differences in the quality of embryos, specifically in category 2, which were higher in the group without *L. salivarius* PS11610 supplementation. This suggests that while *L. salivarius* PS11610 may not affect the number of embryos, it might influence certain aspects of embryo quality.

In the IVF with embryo transfer group, the characteristics of the cases and the primary outcomes were comparable between the two groups (with or without *L. salivarius* treatment). The quality of embryos in categories 2 and 3 was higher in the group without *L. salivarius* PS11610. Despite these differences in embryo quality, the overall outcomes did not show significant improvement with using *L. salivarius* PS11610, indicating that other factors might play a more critical role in the success of IVF with embryo transfer.

A similar study selected 17 couples for assisted reproduction treatment with confirmed urogenital dysbiosis and unknown or idiopathic infertility for a period of 6-month treatment with *L. salivarius* (1 dose every 12 h for females and 1 dose every 24 h for males), prior IVF procedures. Ultimately, *L. salivarius* PS11610 supplementation significantly improve the balance of the urogenital microbiome composition in male and female. Particularly, increasing Lactobacilli percentage in the vagina after 3 and 6 months of treatment. Moreover, the oral intake of *L. salivarius* PS11610 seemed to change the uterine microbiome and improve the general immunological profile of couples, becoming anti-inflammatory. The rates of pregnancy and delivery were also improved [42].

In a study by Sofia Väinämö et al., a beneficial effect of using *Lactobacillus crispatus* was observed in a couple who underwent IVF treatment and fresh embryo transfer [44]. In another study, the importance of a balanced microbiome to a successful IVF procedure was shown, when a cohort study of 84 women who underwent IVF cycles, only 8% of them with bacterial vaginosis, urogenital bacterial imbalance, were able to conceive compared with 40% of clinical pregnancy in women with balanced urogenital microbioma [45]. In a randomized controlled study, Isarin Thanaboonyawat et al. gave intravaginal supplements of *Lactobacillus acidophilus* for 6 days to infertile women significantly lowering the miscarriage rate in the study group compared to the control group, 9.5% and 19.1%, respectively [46].

The most notable findings were observed in the FET group, where the intake of *L. salivarius* PS11610 was associated with a significant increase in live birth (26.4% with *L. salivarius* PS11610 vs. 17.9% without, *p* = 0.034).

In another study, the cervicovaginal environment of women with reproductive failure (repetitive abortion, infertility of unknown origin) was assessed and compared to that of healthy fertile women. Daily oral administration of *L. salivarius* CECT5713 resulted in an overall successful pregnancy rate of 56%. The authors of this study emphasized how probiotics can influence important microbiological, biochemical, and immunological aspects in pregnant women. They concluded that *L. salivarius* could be a promising option for enhancing reproductive success in women experiencing reproductive challenges [47].

Additionally, the rate of biochemical pregnancies, defined as early pregnancies, confirmed by the levels of pregnancy hormones in the blood but not yet visible on an ultrasound, was higher in the *L. salivarius* PS11610 group, achieving statistical significance at a 90% confidence level (*p* = 0.071). This result suggested a positive impact of *L. salivarius* PS11610 on the success rates of FET procedures. In fact, in another study, Irollo AM. et al. showed that the treatment with probiotics and prebiotics in infertile women affected by acute or severe intestinal bacterial imbalance could influence the modulation of some mechanisms involved in embryo implantation [48].

The multiple factor analysis (MFA) further supported these findings by showing that the primary source of variability in the dataset was related to the outcomes rather than the characteristics of the cases. This indicates that the intake of *L. salivarius* PS11610 may enhance FET’s success by improving pregnancy outcomes.

## 5. Conclusions

The administration of *L. salivarius* PS11610 at a dose of 1 × 10^9^ CFU every 12 h at least 1 month before IVF procedures, particularly with FET, appears to enhance the success of these procedures. This enhancement is evidenced by increased live births and biochemical pregnancy rates. While some differences in embryo quality classification were noted, the overall positive impact on pregnancy and birth outcomes underscores the potential of *L. salivarius* PS11610 as a beneficial supplement in assisted reproductive treatments.

Additional research is necessary to investigate how *L. salivarius* PS11610 influences outcomes, particularly concerning its effects on the urogenital microbiome and the immune profiles of couples undergoing IVF. A controlled randomized trial is being prepared to enhance the robustness of findings and provide more conclusive insights.

## Figures and Tables

**Table 1:** Characteristics of IVF procedures without transfer cases during the evaluated periods.

	Without *L. salivarius* PS11610	With *L. salivarius* PS11610	*p*-Value *^,^‡
	(N = 85)	(N = 63)	
**Oocyte extraction Technique**			
Both	2 (2.4%)	0 (0%)	0.702
Cryopreserved	3 (3.5%)	2 (3.2%)	
Punction	80 (94.1%)	61 (96.8%)	
**Oocyte Origin**			
Donor	6 (7.1%)	4 (6.3%)	1.000
Own	79 (92.9%)	59 (93.7%)	
ROPA	0 (0%)	0 (0%)	
**Semen**			
Frozen Testicular Biopsy	2 (2.4%)	4 (6.3%)	0.399
Frozen	2 (2.4%)	3 (4.8%)	
Donor	8 (9.4%)	3 (4.8%)	
Fresh	73 (85.9%)	52 (82.5%)	
**Fertilization Procedure**			
ICSI	50 (58.8%)	39 (61.9%)	0.492
IVF	35 (41.2%)	21 (33.3%)	
**Subject Age**			
Median (Q1–Q3)	37.00 (32.00–39.00)	34.00 (30.00–39.50)	0.219
Min–Max	20.00–45.00	22.00–45.00	

N, number of cases; ROPA, Receiving oocytes from the partner; ICSI, Intracytoplasmic sperm injection; IVF, *In vitro* fertilization. Qualitative variable results are expressed as absolute and relative frequencies (%, over the group margin). Missing values were considered in calculating the relative frequencies, although not evaluated in the comparison. Thus, they were excluded from the table. Quantitative variable results are expressed as median (1st–3rd quartile) and (minimum–maximum). * Kruskal–Wallis test. ‡ Chi-square test.

**Table 2:** Comparison of clinical outcomes obtained from IVF without transfer procedure in the evaluated periods.

	Without *L. salivarius* PS11610	With *L. salivarius* PS11610	*p*-Value *
	(N = 85)	(N = 63)	
**No. of Oocytes**			
Median (Q1–Q3)	11.00 (5.00–20.00)	13.00 (6.50–22.00)	0.429
Min–Max	0.00–48.00	0.00–50.00	
**No. of Frozen Oocytes**			
Median (Q1–Q3)	0.00 (0.00–0.00)	0.00 (0.00–0.00)	0.099
Min–Max	0.00–0.00	0.00–4.00	
**No. Fertilized Oocytes**			
Median (Q1–Q3)	6.00 (2.00–12.00)	7.00 (3.00–11.50)	0.631
Min–Max	0.00–42.00	0.00–43.00	
**No. Embryo**			
Median (Q1–Q3)	6.00 (2.00–11.00)	7.00 (2.50–11.00)	0.510
Min–Max	0.00–38.00	0.00–35.00	
**No. Embryo Quality 1**			
Median (Q1–Q3)	2.00 (0.00–5.00)	2.00 (0.00–4.00)	0.692
Min–Max	0.00–24.00	0.00–17.00	
**No. Embryo Quality 2**			
Median (Q1–Q3)	0.00 (0.00–0.00)	0.00 (0.00–0.00)	0.042
Min–Max	0.00–9.00	0.00–5.00	
**No. Embryo Quality 3**			
Median (Q1–Q3)	0.00 (0.00–0.00)	0.00 (0.00–0.00)	0.133
Min–Max	0.00–1.00	0.00–0.00	
**No. Cryopreserved Embryos**			
Median (Q1–Q3)	3.00 (0.00–5.00)	2.00 (1.00–4.00)	0.522
Min–Max	0.00–24.00	0.00–14.00	
**No. Blastocysts**			
Median (Q1–Q3)	3.00 (0.00–7.00)	3.00 (1.00–7.50)	0.516
Min–Max	0.00–25.00	0.00–27.00	

N, number of cases; IVF, *In vitro* fertilization. Results were also reported as median (1st–3rd quartile) and (minimum–maximum) values. * Kruskal–Wallis test.

**Table 3:** Characteristics of IVF with transfer cases within the evaluated periods.

	Without *L. salivarius* PS11610	With *L. salivarius* PS11610	*p*-Value *^,^‡
	(N = 44)	(N = 32)	
**Oocyte extraction Technique**			1.000
Both	1 (2.3%)	1 (3.1%)	
Cryopreserved	7 (15.9%)	5 (15.6%)	
Punction	36 (81.8%)	26 (81.3%)	
**Oocyte Origin**			0.868
Donor	5 (11.4%)	5 (15.6%)	
Own	38 (86.4%)	26 (81.3%)	
ROPA	1 (2.3%)	1 (3.1%)	
**Semen**			0.428
Frozen Testicular Biopsy	1 (2.3%)	2 (6.3%)	
Frozen	2 (4.5%)	3 (9.4%)	
Donor	2 (4.5%)	3 (9.4%)	
Fresh	39 (88.6%)	24 (75.0%)	
**Fertilization Procedure**			1.000
ICSI	32 (72.7%)	23 (71.9%)	
IVF	11 (25.0%)	9 (28.1%)	
**Subject Age**			
Median (Q1–Q3)	36.50 (33.00–41.00)	39.00 (34.75–43.00)	0.217
Min–Max	18.00–47.00	26.00–48.00	
**No. of Oocytes**			
Median (Q1–Q3)	7.00 (5.00–11.00)	6.50 (3.00–9.25)	0.493
Min–Max	0.00–47.00	1.00–27.00	
**No. Oocytes Fertilized**			
Median (Q1–Q3)	3.00 (2.00–4.25)	3.50 (1.00–7.00)	0.899
Min–Max	0.00–12.00	0.00–21.00	
**No. Embryo**			
Median (Q1–Q3)	3.00 (2.00–4.25)	3.50 (1.00–7.00)	0.702
Min–Max	0.00–11.00	0.00–17.00	
**No, Embryo Quality 1**			
Median (Q1–Q3)	1.00 (0.00–2.00)	1.00 (0.75–3.00)	0.167
Min–Max	0.00–9.00	0.00–7.00	
**No. Embryo Quality 2**			
Median (Q1–Q3)	0.00 (0.00–1.00)	0.00 (0.00–0.00)	0.013
Min–Max	0.00–4.00	0.00–2.00	
**No. Embryo Quality 3**			
Median (Q1–Q3)	0.00 (0.00–0.00)	0.00 (0.00–0.00)	0.050
Min–Max	0.00–2.00	0.00–0.00	
**No. Transferred**			
Median (Q1–Q3)	1.00 (1.00–1.00)	1.00 (0.00–1.00)	0.134
Min–Max	0.00–2.00	0.00–2.00	
**No. Cryopreserved Embryos**			
Median (Q1–Q3)	0.00 (0.00–1.00)	0.00 (0.00–2.00)	0.618
Min–Max	0.00–9.00	0.00–7.00	
**No. Blastocysts**			
Median (Q1–Q3)	2.00 (1.00–3.00)	1.50 (0.00–3.25)	0.621
Min–Max	0.00–9.00	0.00–10.00	

N, number of cases; ROPA, Receiving oocytes from the partner; ICSI, Intracytoplasmic sperm injection; IVF, *In vitro* fertilization. The results of the qualitative variables are expressed as absolute and relative frequencies (%, over the group margin). Missing values were considered when calculating the relative frequencies, although they were not evaluated in the comparison. Thus, they were excluded from the table. The results of the quantitative variables are expressed as median (1st quartile–3rd quartile) and (minimum–maximum) values, which were also reported. * Kruskal–Wallis test. ‡ Chi-square test.

**Table 4:** Comparison of clinical outcomes obtained from IVF with transfer procedure in the evaluated periods.

	Without *L. salivarius* PS11610	With *L. salivarius* PS11610	*p*-Value ‡
	(N = 44)	(N = 32)	
**Biochemical Pregnancy**			1.000
No	28 (63.6%)	21 (65.6%)	
Yes	13 (29.5%)	11 (34.4%)	
**Clinical Pregnancy**			0.327
No	4 (9.1%)	1 (3.1%)	
Yes	9 (20.5%)	10 (31.3%)	
**Miscarriage**			0.350
No	5 (11.4%)	8 (25.0%)	
Yes	4 (9.1%)	2 (6.3%)	
**Live births**			0.350
No	4 (9.1%)	2 (6.3%)	
Yes	5 (11.4%)	8 (25.0%)	

N, number of cases. The results of qualitative variables are expressed as absolute and relative frequencies (%, over the group margin). Missing values were considered in calculating the relative frequencies, although they were not evaluated in the comparison; thus, they were excluded from the table. ‡ Chi-square test.

**Table 5:** Characteristics of IVF with FET cases in the evaluated periods.

	Without *L. salivarius* PS11610	With *L. salivarius* PS11610	*p*-Value *^,^‡
	(N = 235)	(N = 235)	
**Oocyte Origin**			
Donor	28 (11.9%)	36 (15.3%)	0.291
Own	205 (87.2%)	194 (82.6%)	
ROPA	2 (0.9%)	5 (2.1%)	
**Oocytes Type**			
Fresh	216 (91.9%)	219 (93.2%)	0.726
Vitrified	19 (8.1%)	16 (6.8%)	
**Semen**			
Frozen Testicular Biopsy	1 (0.4%)	0 (0%)	0.350
Frozen	20 (8.5%)	16 (6.8%)	
Donor	8 (3.4%)	14 (6.0%)	
Fresh	206 (87.7%)	205 (87.2%)	
**Subject Age**			
Median (Q1–Q3)	37.00 (34.00–41.00)	37.00 (33.00–42.00)	0.831
Min–Max	20.00–50.00	16.00–49.00	
**No. Thawed Embryos**			
Median (Q1–Q3)	1.00 (1.00–2.00)	1.00 (1.00–1.00)	0.213
Min–Max	1.00–16.00	1.00–9.00	
**No. Embryo Transfer**			
Median (Q1–Q3)	1.00 (1.00–1.00)	1.00 (1.00–1.00)	0.270
Min–Max	0.00–2.00	0.00–2.00	
0	8 (3.4%)	6 (2.6%)	0.320
1	175 (74.5%)	189 (80.4%)	
2	52 (22.1%)	40 (17.0%)	
**No. of Days from Thawing to Transfer**			
Median (Q1–Q3)	5.00 (5.00–5.00)	5.00 (5.00–5.00)	0.030
Min–Max	2.00–7.00	2.00–6.00	
Missing	0 (0%)	2 (0.9%)	0.026
2	1 (0.4%)	2 (0.9%)	
3	22 (9.4%)	8 (3.4%)	
4	1 (0.4%)	0 (0%)	
5	174 (74.0%)	176 (74.9%)	
6	36 (15.3%)	47 (20.0%)	
7	1 (0.4%)	0 (0%)	

N, number of cases; ROPA, Receiving oocytes from the partner. The results of the qualitative variables are expressed as absolute and relative frequencies (%, over the group margin). Missing values were considered when calculating the relative frequencies, although they were not evaluated in the comparison. Thus, they were excluded from the table. The results of the quantitative variables are expressed as median (1st quartile–3rd quartile) and (minimum–maximum) values, which were also reported. * Kruskal–Wallis test. ‡ Chi-square test.

**Table 6:** Comparison of clinical outcomes obtained from the FET procedure in the evaluated periods.

	Without *L. salivarius* PS11610	With *L. salivarius* PS11610	*p*-Value ‡
	(N = 235)	(N = 235)	
**Biochemical Pregnancy**			
No	155 (66.0%)	135 (57.4%)	0.071
Yes	80 (34.0%)	100 (42.6%)	
**Clinical Pregnancy**			
No	170 (72.3%)	157 (66.8%)	0.229
Yes	65 (27.7%)	78 (33.2%)	
**No. of sacs**			
0	177 (75.3%)	166 (70.6%)	0.527
1	54 (23.0%)	63 (26.8%)	
2	4 (1.7%)	6 (2.6%)	
**Miscarriage**			
No	218 (92.8%)	219 (93.2%)	1.000
Yes	17 (7.2%)	16 (6.8%)	
**VIP**			
No	234 (99.6%)	235 (100%)	1.000
Yes	1 (0.4%)	0 (0%)	
**Ectopic Pregnancy**			
No	234 (99.6%)	235 (100%)	1.000
Yes	1 (0.4%)	0 (0%)	
**Pregnancy Type**			
Twins	2 (0.9%)	4 (1.7%)	1.000
Single	40 (17.0%)	58 (24.7%)	
**Live Births**			
No	193 (82.1%)	173 (73.6%)	0.034
Yes	42 (17.9%)	62 (26.4%)	

N, number of cases. The results of qualitative variables are expressed as absolute and relative frequencies (%, over the group margin). Missing values were considered in calculating the relative frequencies, although they were not evaluated in the comparison; thus, they were excluded from the table. ‡ Chi-square test.

## Data Availability

The raw data supporting the conclusions of this article will be made available by the authors on request.
